# Individual and community-level factors associated with skilled birth attendants during delivery in Bangladesh: A multilevel analysis of demographic and health surveys

**DOI:** 10.1371/journal.pone.0267660

**Published:** 2022-06-29

**Authors:** Mst. Tanmin Nahar, S. M. Farhad Ibn Anik, Md. Akhtarul Islam, Sheikh Mohammed Shariful Islam

**Affiliations:** 1 Statistics Discipline, Science Engineering & Technology School, Khulna University, Khulna, Bangladesh; 2 Institute for Physical Activity and Nutrition, Deakin University, Melbourne, Victoria, Australia; University of Southern Queensland, AUSTRALIA

## Abstract

**Background:**

Skilled birth attendants (SBAs) play a crucial role in reducing infant and maternal mortality. Although the ratio of skilled assistance at birth has increased in Bangladesh, factors associated with SBA use are unknown. The main goal of our study was to reveal the individual- and community-level factors associated with SBA use during childbirth in Bangladesh. We also showed the prevalence and trend of SBA use and related independent variables in Bangladesh over the past decade.

**Methods:**

This study utilized the Bangladesh Health and Demographic Survey (BDHS) 2017–2018, a cross-sectional study. We used binary logistic regression to examine the extent of variation in SBA use attributable to the individual- and community-level variables.

**Results:**

Overall, 53.35% of women received assistance from SBAs during childbirth. The average annual rate of increase (AARI) in the number of SBA-assisted births over the past 10 years was 8.88%. Respondents who gave birth at or above 19 years had 1.40 times (AOR = 1.40; 95% CI: 1.21–1.62) greater odds of having skilled delivery assistance than respondents aged 18 years old or less. Women and their husband’s education levels were significantly associated with using skilled assistance during delivery, with odds of 1.60 (AOR = 1.60; 95% CI: 1.45–2.01) and 1.41 (AOR = 1.41; 95% CI: 1.21–1.66), respectively compared to those with education up to primary level. Women from rich families and those receiving better antenatal care (ANC) visits were more likely to have professional delivery assistance. Community-level factors also showed significance towards having professional assistance while giving birth. Women from urban communities and those who utilized more than four ANC visits and had completed secondary or higher education showed a greater tendency to use an SBA during childbirth than their counterparts.

**Conclusion:**

The use of SBAs during delivery was significantly associated with some individual- and community-level factors. To reduce maternal and child mortality, there is a need to focus on rural and uneducated people who are less likely to access these facilities. Special programs could increase awareness and help the poor community obtain the minimum facility in maternal care.

## Introduction

Maternal mortality continues to be a considerable challenge for public health systems worldwide. Every day, women and children die from complications related to pregnancy and birth, which are mostly preventable. In 2017, an estimated 0.295 million women died during childbirth, and developing countries like Bangladesh accounted for approximately 44% of this figure [1]. Moreover, Southern Asia has the second-highest maternal mortality rate, leading to the global burden of maternal mortality [2]. Bangladesh’s maternal health had remarkably improved over the past years based on Millennium Development Goal (MDG) targets in 2015 [3–5]. The country is on track to achieve the Sustainable Development Goals (SDGs) [6, 7]. However, according to the MDG 5 progress report [8], Bangladesh performed poorly in the service performance of skilled assistance during delivery, scoring below the center point [5, 9]. Many systematic reviews reported that maternal deaths occur due to obstetric hemorrhage or sepsis, obstructed labor, unsafe abortions, eclampsia, and prenatal and postnatal infections [10–12]. Most of these deaths could have been prevented by ensuring skilled professional services and adequate facilities were available [13].

Professional doctors, nurses, paramedics, midwives, family welfare visitors (FWVs), community skilled birth assistants (CSBAs), and sub-assistant community medical officers (SACMOs) are considered medically trained providers and defined as skilled birth attendants (SBAs) [14]. Conversely, untrained traditional birth attendants (TBAs), and trained TBAs (orthodox village doctors without academic qualifications or uncertified community workers) are considered unskilled birth attendants [14–17]. The skills and proficiency of the person assisting in child delivery and the available healthcare facilities can influence complications during and after delivery [18]. The World Health Organization recommends that every delivery be supervised by an SBA, a healthcare provider who can identify and manage normal labor and birth figure out and provide primary care and referrals [19, 20]. Though there are well-known benefits of high-quality obstetrics care for both mothers and babies, many lower-middle-income countries face large socioeconomic disparities in accessing and using SBA services [21, 22].

Bangladesh has notably improved maternal and newborn health over the past 10 years [23]. The latest Bangladesh Demographic and Health Survey (BDHS) showed that the percentage of SBAs present during deliveries in Bangladesh increased from 21% in 2007 to 53% in 2017–2018 [14]. The fourth Health Population and Nutrition Sector Development Program of the Government of Bangladesh targeted 65% SBA use at delivery by 2022 [14]. To attain this target and increase the presence of SBAs during childbirth in Bangladesh, it is essential to determine the factors that affect the delivery provided by SBAs.

Several factors at different levels impact the utilization of SBAs during delivery [24]. Existing studies in lower-middle-income countries have found that the utilization of SBAs is mainly influenced by a mixture of individual and community characteristics. These studies have found an association between SBA utilization and individual characteristics, such as maternal education [25], gender disparity [26], desire for the pregnancy, birth order [27], and family size [28]. Community-level factors included rural or urban places of residence [29, 30], community education, mass media exposure [31], and antenatal care (ANC) service availability [32]. However, in Bangladesh, a limited number of studies have focused on the factors of SBA utilization at different levels, including the utilization of maternal healthcare services [33, 34]. Therefore, we aimed to examine the individual- and community-level factors associated with the utilization of SBAs during delivery in Bangladesh. This study also fills a knowledge gap, drawing on the multilevel approach to uptake of SBAs in nationally representative data.

## Materials and methods

### Study design and data sources

This study was conducted using the BDHS 2017–2018 data. The BDHS 2017–2018 was conducted under the National Institute of Population Research and Training authority of the Ministry of Health and Family Welfare. A Bangladeshi research firm, Mitra and Associates, carried out the survey [14]. In this survey, a two-stage stratified clustering sampling technique was used. The geographical area of Bangladesh was split into eight administrative divisions. Then, 675 Enumeration Areas (EAs) were selected in the first stage, with 250 urban and 425 rural areas. An EA is a geographic region that is either a collection of smaller towns, a large part of a village, or an entire village with an average of about 120 households. Thus, an EA was the primary sampling unit [14]. In the second stage, 30 households were selected on average from each EA.

The BDHS 2017–2018 included five types of questionnaires. In this study, we used data from the women’s questionnaire. This questionnaire was based on model questionnaires developed for the worldwide Demographic and Health Surveys (DHS)-7 Program, adjusted to the circumstances and requirements in Bangladesh [14]. During this survey, women were asked questions regarding their background characteristics (e.g., age, education, religion, and media exposure), reproductive history, knowledge of uses and sources of family planning methods, ANC, delivery, postnatal care, newborn care, and husband’s background [14]. In our study, we only considered the occasion of the first live birth in a woman’s life. A weighted sample of 4,842 individuals who had had a live birth in the three years preceding the surveys. For women who had had more than one live birth, only the first live birth was considered in this study. For the selected respondent we consider all the delivery places where the birth took place as the home, hospital, clinic, and public, private or non-governmental organizations (NGOs) were included in our study [29, 35–41].

### Outcome variable

The outcome variable was whether an SBA was present while a woman gave birth to her child. In our analysis, we considered doctors, nurses, midwives and community SBAs ‘skilled birth attendants or SBAs’ and recoded these as ‘1’. Other birth attendants, such as TBAs, relatives, NGO workers and neighbours, were considered ‘unskilled birth attendants’ and recoded as ‘0’. This classification was based on previous studies in other countries [29, 42]. Thus, the SBA is a binary variable with two mutually exclusive and exhaustive categories.

### Independent variables

In this multilevel analysis, we utilised both individual- and community-level variables as independent variables. Individual-level factors include the respondents’ current age, religion, occupation, division, employment status, education status, access to mass media, birth order, participation in healthcare decisions and ANC visits. Further, their husbands’ employment and education status and whether they wanted the pregnancy while pregnant were also considered. These variables were selected based on previous literature from developing countries [25, 29, 39, 40, 43–46].

Some variables were recoded for better interpretation. Women who had not watched TV, listened to the radio or read a newspaper at least once a week were categorized as ‘no access’, while others were grouped as ‘have access’. The variables ‘healthcare decision’ and ‘purchase decision’ were based on whether the respondent had participated in her healthcare or household purchases. Women who exclusively or mutually with a male partner had last say on their medical care choice were categorized as ‘participation’; otherwise, they were categorized as ‘no participation’. Moreover, ANC visits were categorized as ‘no ANC’, ‘1 to 8 ANC visits’ and ‘more than 8 ANC visits’. We also recoded religion (Islam, others), body mass index (BMI) (normal, not normal) and desire for pregnancy (yes, no more) into binary categories. When a woman became pregnant, a wanted pregnancy was categorized as ‘yes’ if the respondent wanted their pregnancy then or later, and ‘no more’ when they did not want pregnancy anymore. The wealth index was recoded by identifying ‘poorest’ and ‘poorer’ as ‘poor’, and ‘richest’ and ‘richer’ as ‘rich’, while the middle wealth category remained the same. Occupation was coded as ‘working’ and ‘not working’, while husband’s occupation was divided into two categories—‘farming and labour-intensive’ and ‘job or business’. Lastly, the education level of the respondents and their husbands were coded as ‘up to primary’ and ‘secondary and above’.

Five community-level factors were considered in this analysis. These are the residence (rural or urban), community wealth (concentration of households classified as rich or poor in a cluster), community media (concentration of women exposed to the media in a cluster), community ANC visits (concentration of women going for at least four ANC visits during her pregnancy in a cluster), community education (concentration of educated women in a cluster) and community healthcare distance (concentration of women who thought the distance was a problem in seeking healthcare in a cluster). These variables were created by aggregating selected categories to the clusters and dividing them into suitable categories based on the previous literature if no such variables were listed in the BDHS 2017–2018 [29, 40].

### Statistical analysis

We conducted a univariate analysis to observe the frequency of the selected variables. In the bivariate analysis, the changes in associate variables were shown for the cross-tabulation outcome variable. The chi-square value of these variables was also demonstrated in this analysis. We also checked the average annual rate of increase (AARI) of four consecutive BDHS data, from 2007 to 2017–2018. For this calculation, we used the formula, *Y*_*t*+*n*_ = *Y*_*t*_((1+*r*)*n*) for skilled delivery assistance, where *Y*_*t*_ = prevalence of skilled delivery assistance, *r* = annual rate of change, *n* = number of years between two surveys and *Y*_*t*+*n*_ = prevalence of skilled delivery assistance of the (*t* + *n*)^*th*^ year, which was adjusted and adopted, utilizing the information given in the UNICEF technical note [47].

In the multilevel analysis, the fixed effect and random effect binary logistic regressions were used to examine the extent of variation in the SBA attributable to individual- and community-level variables. This model is more appropriate for hierarchically structured data such as DHS data [48]. We first constructed an empty model that only used a random intercept to conduct this analysis. This estimated the degree of correlation in the skilled delivery assistance at cluster (community) levels. Then, we included all the individual-level factors in the second model to observe their effect on professional delivery assistance. Finally, we added the community-level characteristics in the third model to observe which contextual factors influenced the use of skilled delivery assistance. Then, the model was specified as:

$$log\left(\frac{\pi_{ij}}{{1-\pi }_{ij}}\right)=Y_{ij}=\beta_{0}+\beta_{1}X_{1ij}+\cdots +\beta_{n}X_{nij}+e_{ij}\;where,e_{ij}∼N(0,\sigma^{2})$$

In this equation, *Y*_*ij*_ = outcome variable for individual *i* in *j* group, *X*_*ij*_ = individual-level variable for the *i*^*th*^ individual in group *j* and *e*_*ij*_ = individual-level error assumed independent and normally distributed with mean zero and a variance of *σ*^2^. This model evaluated relationships and variances at multiple levels [48]. The fixed effects were computed by the odds ratio of binary logistic regression, and the random effects were estimated by the intra-class correlation (ICC). ICC was calculated as $\frac{\tau }{\tau +(\frac{\pi^{2}}{3})}$, where *τ* is the community-level variance [49]. In this study, all the analyses were performed using Stata version 14.2.

## Results

The univariate analysis from Table 1 revealed that, overall, 53.35% of women utilized SBAs. About 65.84% of the women lived in rural areas, and the majority (91.45%) were Muslim women. More than one-third of the women (34.01%) and nearly half (47.65%) of their husbands reported their education level as ‘up to primary’ level. In this research, more than half (55.82%) of the women gave birth to their first child at the age of 18 or below, and nearly two-thirds of the women (60.35%) were employed. During the survey, more than one-third of their husbands (41.88%) were engaged in farming and labour-intensive. Moreover, a high percentage of the women (41.97%) were in the poorest wealth quantile. Nearly two-thirds of the women had access to the mass media, and about 30% of them did not partake in their healthcare choice. However, 85.42% of the women reported having one to eight ANC visits before their most recent deliveries, and most of the women gave birth to more than one child. The women included in the study mostly wanted to be pregnant (91.76%) and mostly had normal BMI (61.57%). The analysis also showed the percentage of community-level factors. Women were living in communities where more than half of the affluent households (61.81%), secondary and higher educated (54.42%), access to media (69.83%), and slightly only 49.98% reported using ANC visits.

**Table 1:** Weighted frequency distribution for selected variables.

Variables	Categories	Frequency	Percentage (%)
Residence	Rural	3188	65.84
	Urban	1654	34.16
Religion	Islam	4428	91.45
	Others	414	8.55
Respondent education	Up to primary	1647	34.01
	Secondary and above	3195	65.99
Age at first birth	Less or equal 18	2703	55.82
	Greater or equal 19	2139	44.18
Respondent occupation	Not working	2922	60.35
	working	1920	39.65
Husband occupation	Farming, labour-intensive and others	2028	41.88
	Job and business	2814	58.12
Wealth index	Poorest	2032	41.97
	Middle	877	18.11
	Rich	1933	39.92
Husband education	Up to primary	2307	47.65
	Secondary and above	2535	52.35
Skilled birth attendants	Unskilled	2259	46.65
	Skilled	2583	53.35
Media exposure	No	1732	35.77
	Yes	3110	64.23
Birth order	More than one	3012	62.21
	One	1830	37.79
ANC visits	No ANC visits	388	8.01
	1 to 8 ANC visits	4136	85.42
	More than 8 ANC visits	318	6.57
Health care choice	Husband or others	1318	27.22
	Respondent alone or respondent and husband	3524	72.78
BMI	Normal	2981	61.57
	Not normal	1861	38.43
Wanted pregnancy	No more	399	8.24
	Yes	64443	91.76
Community wealth	Poor	1849	38.19
	Rich	2993	61.81
Community media exposure	No	1461	30.37
	Yes	3381	69.83
Community ANC	No	2422	50.02
	Yes	2420	49.98
Community education	Up to primary	2207	45.58
	Secondary and above	2635	54.42

Table 2 shows the percentage distribution of bivariate association of individual and community characteristics for women’s chosen predictors according to SBAs. The selected individual- and community-level factors were statistically significant (*p* = 0.000) with the utilization of women’s SBAs.

**Table 2:** Percentage distribution of individual and community characteristics for the selected predictors of women according to the SBAs, using the χ2 test (bivariate association).

Variable	Skilled Birth Attendant		χ2 test	P-value
	Unskilled Count (%)	Skilled Count (%)		
**Residence**				
Rural	1707 (35.25)	1481 (30.59)	178.03	0.000
Urban	552 (11.40)	1102 (22.76)		
**Religion**				
Islam	2106 (43.49)	2322 (47.96)	17.11	0.000
Others	153 (3.16)	261 (5.39)		
**Respondent education**				
Up to primary	1104 (22.80)	543 (11.21)	416.41	0.000
Secondary and above	1155 (23.85)	2040 (42.13)		
**Age at first birth**				
Less or equal 18	1485 (30.67)	1218 (25.15)	168.74	0.000
Greater or equal 19	774 (15.99)	1365 (28.19)		
**Respondent occupation**				
Not working	1058 (21.85)	862 (21.85)	91.28	0.000
Working	1201 (24.80)	1721 (35.54)		
**Husband occupation**				
Farming, labour-intensive, and others	1150 (23.75)	878 (18.13)	141.67	0.000
Job and business	1109 (22.90)	1705 (22.90)		
**Wealth index**				
Poorest	1347 (27.82)	685 (14.15)	642.12	0.000
Middle	407 (8.41)	470 (9.71)		
Rich	505 (10.43)	1428 (29.49)		
**Husband education**				
Up to primary	1422 (29.37)	885 (18.28)	397.53	0.000
Secondary and above	837 (17.29)	1698 (35.07)		
**Media exposure**				
No	1119 (23.11)	613 (12.66)	349.22	0.000
Yes	1140 (23.54)	1970 (40.69)		
**Birth order**				
More than one	1627 (33.60)	1385 (28.60)	173.60	0.000
One	632 (13.05)	1198 (24.74)		
**ANC visits**				
No ANC visits	334 (6.90)	54 (1.12)	332.91	0.000
1 to 8 ANC visits	1859 (38.39)	2277 (47.03)		
More than 8 ANC visits	66 (1.36)	252 (5.20)		
**Health care choice**				
Husband or others	643 (13.28)	675 (13.94)	3.31	0.069
Respondent alone or respondent and husband	1616 (33.37)	1908 (39.41)		
**BMI**				
Normal	1500 (30.98)	1481 (30.59)	41.85	0.000
Not normal	759 (15.68)	1102 (22.76)		
**Wanted pregnancy**				
No more	254 (5.25)	145 (2.99)	50.52	0.000
Yes	2005 (41.41)	2438 (50.35)		
**Community wealth**				
Poor	1189 (24.56)	660 (13.63)	374.45	0.000
Rich	1070 (22.10)	1923 (39.71)		
**Community media exposure**				
No	988 (20.40)	473 (9.77)	369.71	0.000
Yes	1271 (26.25)	2110 (43.58)		
**Community ANC**				
No	1431 (29.55)	991 (20.47)	300.80	0.000
Yes	828 (17.10)	1592 (32.88)		
**Community education**				
Up to primary	1371 (28.31)	836 (17.27)	389.79	0.000
Secondary and above	888 (18.34)	1747 (36.08)		

The AARI of our dependent variable, SBA use or presence during delivery, from 2007 to 2017–2018 was 8.88%. Table 3 shows the AARI in SBA use during delivery of different independent variables from 2007 to 2017–2018 in Bangladesh. Rural women reported a higher (13.28%) AARI of the SBAs, comparably urban women. There was a remarkable change in the average annual rates of qualified assistance during delivery among Muslim women (9.04%) compared to women of other religions. The AARI in SBA use increased with the women and their husbands’ education levels. Between 2007 and 2017–2018, the total AARI in SBA use was highest (8.91%) among women who reported their education level as secondary and above. The total AARI in SBA use was also the highest (11.60%) among women whose husbands had received at least ‘up to primary’ level of education.

**Table 3:** The average annual rate of increase in the utilization of SBAs during delivery in Bangladesh, according to background characteristics in the 2007 to 2017–2018 BDHS data.

Variable	2017		2014		2011		2007	
	Skilled delivery assistant (%)	Percent AARI (2014–2017)	Skilled delivery assistant (%)	Percent AARI (2011–2014)	Skilled delivery assistant (%)	Percent AARI (2007–2011)	Skilled delivery assistant (%)	Percent AARI (total, 2007–2017)
**Residence**								
Rural	30.59	1.22	29.50	22.59	16.01	16.17	8.79	13.28
Urban	22.76	15.69	14.70	-3.35	16.28	3.84	14.00	4.98
**Religion**								
Islam	47.96	5.95	40.32	13.26	27.75	8.28	20.19	9.04
Others	5.39	10.64	3.98	-4.29	4.54	14.84	2.61	7.52
**Respondent education**								
Up to primary	11.21	1.75	10.64	10.06	7.98	13.32	4.84	8.76
Secondary and above	42.13	7.76	33.67	11.47	24.31	7.88	17.95	8.91
**Age at first birth**								
Less or equal 18	25.15	4.51	22.03	12.46	15.49	12.24	9.76	9.93
Greater or equal 19	28.19	8.16	22.28	9.87	16.80	6.56	13.03	8.02
**Respondent occupation**								
Not working	21.85	-	-	-	0.25	- 65.66	17.97	1.98
Working	35.54	-	-	-	31.41	59.78	4.82	22.12
**Husband occupation**								
Farming, labour-intensive and others	18.13	8.23	14.30	10.36	10.64	10.10	7.24	9.61
Job and business	22.90	-8.62	30.01	11.49	21.65	8.63	15.55	3.95
**Wealth index**								
Poorest	14.15	13.77	9.61	20.44	5.50	22.53	2.44	19.22
Middle	9.71	6.23	8.10	16.90	5.07	19.82	2.46	14.72
Rich	29.49	3.49	26.60	6.99	21.72	4.97	17.89	5.13
**Husband education**								
Up to primary	18.28	5.43	15.60	14.25	10.46	14.43	6.10	11.60
Secondary and above	35.07	6.89	28.71	9.56	21.83	6.94	16.69	7.71
**Media exposure**								
No	12.66	9.36	9.68	24.06	5.07	12.28	3.19	14.78
Yes	40.69	5.52	34.63	8.36	27.22	8.54	19.61	7.57
**Birth order**								
More than one	28.60	8.57	22.35	9.08	17.22	10.33	11.62	9.42
One	24.74	4.05	21.96	13.37	15.07	7.77	11.17	8.28
**ANC visits**								
No ANC visits	1.12	-27.59	2.95	-44.46	17.22	73.51	1.90	-5.15
1 to 8 ANC visits	47.03	6.28	39.18	14.04	26.42	9.36	18.47	9.79
More than 8 ANC visits	5.20	33.82	2.17	-3.57	2.42	0.00	2.42	7.95
**Health care choice**								
Husband or others	13.94	-20.49	27.74	34.35	11.44	-7.42	15.57	-1.09
Respondent alone or respondent and husband	39.41	33.51	16.56	-7.39	20.85	30.36	7.22	18.49
**BMI**								
Normal	30.59	6.63	25.23	11.69	18.11	16.53	9.82	12.03
Not normal	22.76	6.05	19.08	10.39	14.18	2.25	12.97	5.78
**Wanted pregnancy**								
No more	2.99	-0.77	3.06	6.27	2.55	2.84	2.28	2.75
Yes	50.35	6.88	41.24	11.51	29.74	9.72	20.52	9.39

Further, working women reported utilizing SBAs (22.12%) more than not working women (1.98%). In contrast, their husbands who were engaged in farming and labour-intensive work were more conscious (9.61%) about SBA use over the 10 years than those who categorised their work as ‘job or business’ (3.95%). During the birth of their first child, women aged 18 or younger reported a slight increase (9.93%) in the use of attending SBAs. The overall AARI in SBA use also increased in women who wanted their pregnancy. Women with birth order more than one was also slightly increased in SBAs (9.42%). Women alone or women and their husbands or partners who made decisions on healthcare had a higher rate of increase in SBA use (18.49%). There was a decrease in the rate of SBA use in women who had no ANC check-ups (5.51%), but there was an increase (9.79%) in those who had one to eight ANC check-ups. Women who had no access to media reported a higher increase in SBA use (14.78%), and they mostly had a normal BMI (12.03%). The changes in SBA use were greatest among low-income families (19.22%).

The multilevel model analysis results from Table 4 show the SBAs of women in Bangladesh, according to 2017–2018 BDHS data. The intercept or null model (Model 0) revealed an ICC of 27.5%. This means that approximately 27.5% of the variance in the outcome influences the delivery assistance operated at the community level. This ICC value is good enough (as the ICC value is more significant than zero) to deal with the multilevel modelling in this case [50]. Similarly, this null model (Model 0) depicted statistically substantial variability in the odds of SBA utilisation between communities (p-value = 0.000). Model 1 indicates that only individual-level predictors—age at first birth, the respondent’s education, their husband’s education, media exposure, wealth index, birth order, BMI, the respondent’s occupation, their husband’s occupation, religion, ANC visits, healthcare choice and desire for the pregnancy—were significantly associated with SBA utilisation. The ICC in Model 1 also indicated that 12.6% of women’s delivery assistance variation was attributable to vary across the community level.

**Table 4:** Multilevel binary logistic regression analysis of individual- and community-level factors in SBA use among women in Bangladesh, 2017–2018 DHS.

	Model 0^a^	Model 1^b^		Model 2^c^		Model 3^d^	
		AOR (95% CI)	P value	AOR(95% CI)	P value	AOR(95% CI)	P value
**Fixed Effects Individual-Level Factors**							
**Age at first birth**							
Less or equal 18 ^ref^		1	0.000			1	
Greater or equal 19		1.40 (1.21–1.61)				1.40 (1.21–1.62)	0.000
**Respondent education**							
Up to primary ^ref^		1				1	
Secondary and above		1.71 (1.45–2.01)	0.000			1.60 (1.36–1.89)	0.000
**Husband education**							
Up to primary ^ref^		1				1	
Secondary and above		1.43 (1.22–1.67)	0.000			1.41 (1.21–1.66)	0.000
**Media exposure**							
No ^ref^		1				1	
Yes		1.4 (1.21–1.67)	0.000			1.21 (1.02–1.43)	0.027
**Wealth index**							
Poorest ^ref^		1				1	
Middle		1.40 (1.15–1.71)	0.001			1.26 (1.03–1.53)	0.025
Rich		2.67 (2.20–3.23)				2.05 (1.67–2.52)	0.000
**Birth order**							
More than one ^ref^		1				1	
One		1.67 (1.44–1.94)	0.000			1.68 (1.44–1.96)	0.000
**BMI**							
Normal ^ref^		1				1	
Not normal		1.32 (1.14–1.53)	0.000			1.30 (1.12–1.50)	0.000
**Respondent occupation**							
Not working ^ref^		1				1	
Working		1.27 (1.09–1.48)	0.002			1.25 (1.07–1.45)	0.004
**Religion**							
Islam ^ref^		1				1	
Others		1.45 (1.09–1.91)	0.010			1.42 (1.08–1.87)	0.012
**ANC visits**							
No ANC visits ^ref^		1				1	
1 to 8 ANC visits		3.94 (2.83–5.49)	0.000			3.55 (2.55–4.94)	0.000
More than 8 ANC visits		8.81 (5.59–13.86)	0.000			6.89 (4.37–10.86)	0.000
**Husband occupation**							
Farming, labour-intensive and others ^ref^		1				1	
Job and business		1.16 (1.00–1.35)	0.046			1.16 (1.00–1.35)	0.046
**Health care choice**							
Husband or others ^ref^		1				1	
Respondent alone or respondent and husband		1.21 (1.03–1.41)	0.020			1.17 (1.00–1.37)	0.050
**Wanted pregnancy**							
No more ^ref^		1				1	
Yes		1.38 (1.06–1.80)	0.018			1.44 (1.10–1.87)	0.008
**Community Level Factors**							
**Residence**							
Rural ^ref^				1		1	
Urban				1.47 (1.22–1.78)	0.000	1.17 (0.96–1.43)	0.121
**Community wealth**							
Poor ^ref^				1		1	
Rich				1.52 (1.22–1.90)	0.000	1.14 (0.90–1.44)	0.271
**Community media exposure**							
No ^ref^				1		1	
Yes				1.63 (1.30–2.05)	0.000	1.33 (1.05–1.68)	0.017
**Community ANC visits**							
Below 4 ^ref^				1		1	
4 and above				1.96 (1.65–2.33)	0.000	1.52 (1.27–1.82)	0.000
**Community education**							
Up to primary ^ref^				1		1	
Secondary and above				1.93 (1.59–2.35)	0.000	1.51 (1.24–1.85)	0.000
**Measures of variation for random effect estimates for skilled assistance delivery**							
**Estimates**	**Model 0** ^**a**^	**Model 1** ^**b**^		**Model 2** ^**c**^		**Model 3** ^**d**^	
Community-level variance (SE)	1.26* (0.13)	0.47* (0.08)		0.42* (0.07)		0.36* (0.07)	
Explained Variation^i^ (PCV)	-	62.69		66.66		71.43	
ICC (%)	27.5%	12.6%		11.2%		9.7%	
Log likelihood	-3129.20	-2691.83		-2954.12		-2644.22	
AIC	6262.40	5417.67		5922.24		5332.44	

Key: ^ref^ Reference, ^AOR^ Adjusted Odds Ratio, ^CI^ Confidence Interval, ^SE^ Standard Error, ^ICC^ Intraclass Correlation Coefficient, ^AIC^Akaiki Information Criteria, ^PCV^ Percentage Change in Variance

^a^Intercept or null model

^b^ Model includes only individual-level predators

^c^ Includes only Community-level predictors

^d^ Full model includes significant individual and community-level predictors

^i^ Compared with the intercept or null model

*p-value = 0.000

Similarly, five community-level predictors were included in Model 2. These—including residence, community wealth level, community media exposure, community ANC visits and community education level—significantly impacted SBA use during delivery. The ICC of Model 2 also suggested an 11.2% variation in women’s delivery assistance between the communities. Model 3 (full model) combined both individual- and community-level predictors, and the random slop effects depicted a total of 71.43% variation compared with the null Model 0.

The multilevel analysis model depicted that women aged 19 or above at first birth were 40% (AOR = 1.40; 95% CI: 1.21–1.62) more likely to utilise SBAs during delivery than those aged 18 or below. The women and their husband’s education levels were both significantly associated with SBA use. Women with secondary-level education and above had greater odds (AOR = 1.60; 95% CI: 1.36–1.89) of using SBAs than with primary level education only. Similarly, women’s husbands who attained secondary-level education and above were 41% (AOR = 1.41; 95% CI: 1.21–1.66) more likely to use SBAs than those with primary level education. Regarding media exposure, women who had access to the mass media were 1.21 times more likely (AOR = 1.21; 95% CI: 1.02–1.43) to utilise SBAs during delivery than women who had no access to the mass media. Both women and their husband’s occupations were significant factors influencing the use of SBAs. Working women were 1.25 times more likely to use SBAs (AOR = 1.25; 95% CI: 1.07–1.45) than women who were not working. Women’s partners or husbands who had jobs or businesses had greater odds (AOR = 1.16; 95% CI: 1.00–1.35) or were16% more likely to utilise SBAs during delivery than those whose husbands were doing farming and labour-intensive work. In the case of the wealth index, women from affluent households had 105% greater odds (AOR = 2.05; 95% CI: 1.67–2.52), and women from middle families had 25% greater odds (AOR = 1.25; 95% CI: 1.07–1.45) of using SBAs compared to women from poor households. Regarding the birth order, women with the first birth order were 68% (AOR = 1.68; 95% CI: 1.44–1.96) more likely to use SBAs than others. Although the percentage of Muslim women was higher in our research, other religious women were 1.42 times (AOR = 1.42: 95% CI: 1.08–1.87) more likely to be conscious of using SBAs than Muslim women. Women alone or women and their husbands who made healthcare decisions were 1.17 times more likely to use SBAs (AOR = 1.17; 95% CI: 1.00–1.37) than those who relied only on their husbands or others for healthcare decisions. Further, women who were willing to be pregnant had 1.44 times greater odds (AOR = 1.44; 95% CI: 1.10–1.87) of using SBAs than women who were unwilling to be pregnant. Women who had one to eight ANC check-ups were 3.55 times more likely (AOR = 3.55; 95% CI: 2.55–4.94) to use SBAs than women who had no ANC visits. Similarly, women who had more than eight ANC visits had greater odds or were about 6.89 times (AOR = 6.89; 95% CI: 4.37–10.86) more likely to obtain SBA services than women who had no ANC visits.

The results of the community-level predictors revealed that community media exposure, community ANC visits and community education level were significant in relation to the use of SBAs. Women in communities with access to the mass media were 33% more likely (AOR = 1.33; 95% CI: 1.05–1.68) to use SBAs during delivery than women in communities with no media exposure. Likewise, women in the community who had four or more ANC visits were almost two times more likely to utilize SBAs during delivery (AOR = 1.52; 95% CI: 1.27–1.82) than the women from the cluster who had below four ANC visits. Women in communities who attained secondary-level education and above were almost twice as likely (AOR = 1.52; 95% CI: 1.24–1.85) to use SBAs compared to women who attained up to the primary level of education only.

## Discussion

This study aimed to assess the effect of the individual- and community-level characteristics on skilled assistance during delivery care in Bangladesh. The annual average rate of delivery by SBAs over the past 10 years showed how individual-level factors changed in their influence on SBA use. Our analysis detected a 27.5% contribution of unobserved community-level factors influencing SBA use during delivery.

Our findings show that religion is positively associated with the practice of SBA use. Similar to previous studies, our results show that Muslim women were less likely to use SBA care than non-Muslim women [51]. This might be due to traditional beliefs, a conservative culture, subjects and related practices that have influenced these women or their husbands to not seek a delivery outside the home or obtain maternal healthcare services [52–54]. This study also found that the level of education of the respondents and their husbands was positively associated with SBA assistance during a recent delivery. This finding aligns with previous studies in Bangladesh and elsewhere that described a strong agreement between education and professional delivery assistance and other maternal health services [55–59]. Improvements in the women and their husbands’ educational achievement increased the likelihood of seeking SBA care. This may enhance participation in decision-making through economic independence and autonomy, intensifying health-seeking behavior and expanding social capital through the enlargement of social interfaces. Well-educated women may influence their neighbors and relatives with an educated perception, which would expose them to women who want to be more active in deciding on SBA care services during delivery [60]. However, both educated women and their husbands may better obtain health messages and build attitudes towards skilled assistance during delivery through the mass media [39].

This study also showed the association between occupation and the likelihood of engaging SBA services during delivery. Moreover, women’s working status positively impacted the utilization of SBAs during delivery. Similarly, the high prevalence of skilled care for delivery was observed if their husbands were engaged in a professional job or business. This same scenario was revealed in other studies [61]. The high rates of women unemployment and their husbands’ low income and poverty might prevent utilizing SBAs during delivery. Similar to earlier studies in Bangladesh [33, 35, 62] and other countries [63], our findings show that women with a higher wealth index had greater odds of utilizing SBAs during delivery. A possible reason for this may be that women from poor households may not have adequate funds for the added expense of engaging an SBA [62, 64]. However, increasing women’s economic empowerment can improve maternal health by using skilled personnel for child delivery.

The birth order was found to be significantly associated with SBA use. Women with the first birth order were more concerned about using an SBA than others. Women’s perceptions may be a factor here, and a study found that nearly 50% of women consider their first pregnancy riskier than subsequent pregnancies [27]. Similarly, women with more ANC visits had higher expectations of using SBAs, which is consistent with previous studies [45]. This suggests that women might have learned the importance of using SBAs during delivery from their ANC visits. Previous studies have found that ANC visits emphasize professional maternal delivery services [55, 65–67]. Greater community utilisation of ANC visits showed that people were more conscious of using SBAs than TBAs. Surprisingly, we found that the utilisation of SBAs for delivery was consistent with women who wanted a child when they became pregnant. Conversely, an earlier inquiry showed no significant association between SBA use and women wanting to be pregnant [45]. At their first baby’s birth, women aged 19 years or older were more aware of utilising SBAs during delivery. A possible explanation for this is that they may have had more time for education and had received counselling from community health providers [68], who recommended SBAs [69]. The individual predictor, access to media, and women from high concentration community mass media accessed also showed a significant association with the use of SBA care. A previous study found an effective measure between media exposure and SBA care [30]. A possible explanation may be that watching TV, reading the newspapers and listening to the radio have motivated them to opt for delivery assisted by qualified providers, which might encourage safe and skilled delivery practices to reach the community.

The findings from this study using the individual-level predictor model revealed that women from rural areas were less likely to use SBAs during delivery than their counterparts in urban areas. Previous studies have reported similar findings, where rural mothers were less likely to utilize SBAs [35, 70, 71]. This can likely be attributed to the unavailability of healthcare facilities in rural areas and the difficulties of travelling to healthcare clinics or hospitals or making appointments with medical personnel.

## Strengths and limitations

This research was done based on nationally representative data measured using tools that are acknowledged and approved worldwide. This study identified several factors, both individual- and community-level indicators, that influence the effects of using SBAs during delivery in Bangladesh. This study also showed the AARI in SBA use, using BDHS data published over the past 10 years. Further, the cross-sectional nature of the sample data was gathered retrospectively and tended to provide the desired information when estimating the results. No causality can be introduced thoroughly as the condition may change for the better even after the birth of a child. Still, this study has some limitations. For instance, we could not include and examine several factors related to the utilization of SBAs, such as the transportation system, distance from the healthcare facility, geographical areas, delivery cost and other medical conditions.

## Conclusion

This study identified individual- and community-level variables associated with the use of SBAs during childbirth in Bangladesh. Our findings suggest that individual-level factors like age, education, husband’s education, wealth index, husband and respondent’s occupation, and ANC visits significantly impact SBA use. Community-level variables such as community residence, media access, ANC visit, education and wealth have a significant association with SBA use during delivery. Respondents from a highly educated cluster, a rich family cluster and those that utilised four or more ANC visits and had a high level of media exposure tended to utilise SBA services more than those from other clusters. The findings of this study have important implications for informing the government, health planners and other public health stakeholders to alleviate the socioeconomic inequalities in SBA services in Bangladesh. Education, awareness, increasing media access and monetary help for the poor might be key to boosting the utilization of SBA services. Additionally, organising awareness programs based on factors associated with SBAs might prevent maternal and child deaths in Bangladesh.

## Supporting information

S1 Table Multicollinearity test. (DOCX)

## List of abbreviations

SBAs: Skilled Birth Attendants
AARI: Average Annual Rate of Increase
BMI: Body Mass Index, ref = Reference
AOR: Adjusted Odds Ratio
CI: Confidence Interval
SE: Standard Error
ICC: Intraclass Correlation Coefficient
AIC: Akaiki Information Criteria
PCV: Percentage Change in Variance
NGO: Non-Governmental Organization
