# Supplementary material for: Individual and community-level factors associated with skilled birth attendants during delivery in Bangladesh: A multilevel analysis of demographic and health surveys
Source: PLoS One. 2022 Jun 29;17(6):e0267660. doi: 10.1371/journal.pone.0267660 (PMC9242462; doi:10.1371/journal.pone.0267660)
Supplement: S1 Table — (DOCX) [file pone.0267660.s001.docx]

Table 1: Multicollinearity checking

| **Variable** | **VIF** | **1/VIF** |
| --- | --- | --- |
|  |  |  |
| **Residence** | | |
| Rural | 1.00 | 0.998 |
| Urban |  |  |
| **Respondent education** | | |
| Up to primary | 1.00 | 0.998 |
| Secondary and above |  |  |

**Comment**: Both Variance inflation factor (VIF) values are 1, which is less than 5, which indicates that multicollinearity is not a problem for this model.

Table 2: Multicollinearity checking

| **Variable** | **VIF** | **1/VIF** |
| --- | --- | --- |
|  |  |  |
| **Religion** | | |
| Islam | 1.55 | 0.644 |
| others |  |  |
| **Age at first birth** | | |
| Less or equal 18 | 1.10 | 0.907 |
| Greater or equal 19 |  |  |
| **Respondent occupation** | | |
| Not working | 1.08 | 0.925 |
| working |  |  |
| **Husband occupation** | | |
| Farming, labor, and others | 1.12 | 0.890 |
| Job and business |  |  |
| **Wealth index** | | |
| Poorest | 1.55 | 0.644 |
| Middle |  |  |
| rich |  |  |
| **Husband education** | | |
| Up to primary | 1.40 | 0.714 |
| Secondary and above |  |  |
| **Media exposure** | | |
| No | 1.29 | 0.774 |
| Yes |  |  |
| **Birth order** | | |
| More than one | 1.14 | 0.881 |
| One |  |  |
| **ANC visits** | | |
| No ANC visits | 1.10 | .909 |
| 1 to 8 ANC visits |  |  |
| More than 8 ANC visits |  |  |
| **Health care choice** | | |
| Husband or others | 1.02 | 0.981 |
| Respondent alone or respondent and husband |  |  |
| **BMI** | | |
| Normal | 1.01 | 0.983 |
| Not normal |  |  |
| **Wanted pregnancy** | | |
| No more | 1.07 | 0.932 |
| Yes |  |  |

**Comment:** From table 2, VIF values are below 5, which indicates that multicollinearity is no longer a problem in the model. A value between 1 and 5 indicates a mild correlation between a given independent variable and other independent variables in the model, which is not severe enough to require consideration.
